# Supplementary material for: IGF2 Mediates Resistance to Isoform-Selective-Inhibitors of the PI3K in HPV Positive Head and Neck Cancer
Source: Cancers (Basel). 2021 May 7;13(9):2250. doi: 10.3390/cancers13092250 (PMC8125641; doi:10.3390/cancers13092250)

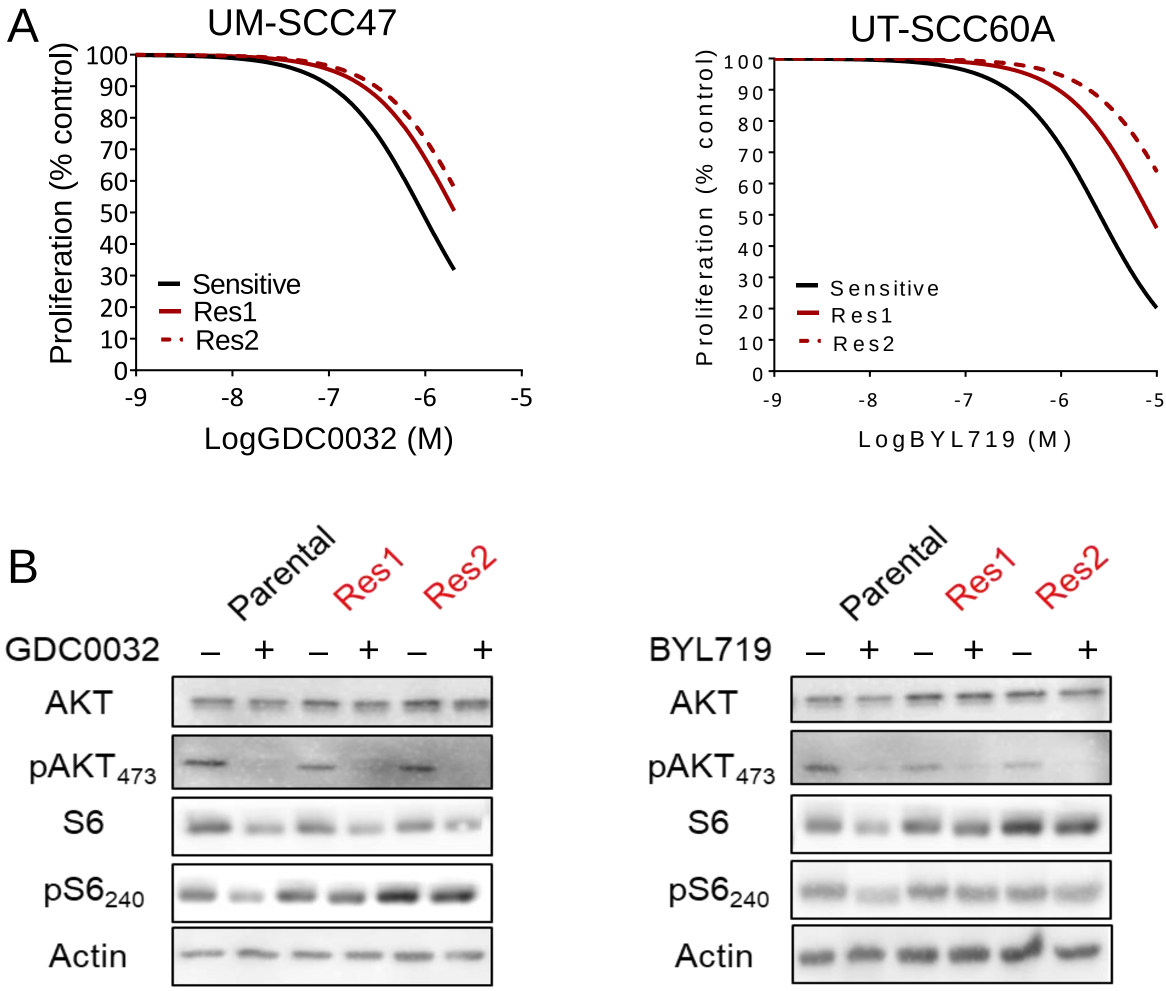

Figure S1: Characterization of UM-SCC47 and UT-SCC60A sensitive and resistant cells. **(A)** Dose-response curve of UM-SCC47 and UT-SCC60A sensitive and resistant cell (res1 and res2) following 4-days of treatment with increasing concentration of GDC0032 (0-2 μM) and BYL719 (0-10 μM), respectively. **(B)** UM-SCC47 and UT-SCC60A sensitive and resistant cells treated for 24 h with DMSO, GDC0032 (500 nM) or BYL719 (2 μM), respectively, after which cells were lysed. The indicated proteins and phosphorylated proteins were measured using western blot.

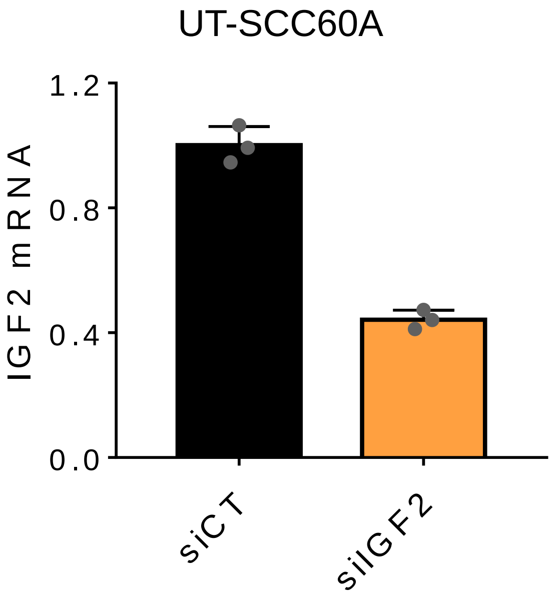

Figure S2: IGF2 silencing using siRNA. RT-PCR analysis measuring IGF2 mRNA levels in sensitive UT-SCC60A following siRNA transfection with scramble RNAi (siCT) or RNAi against IGF2 (siIGF2)

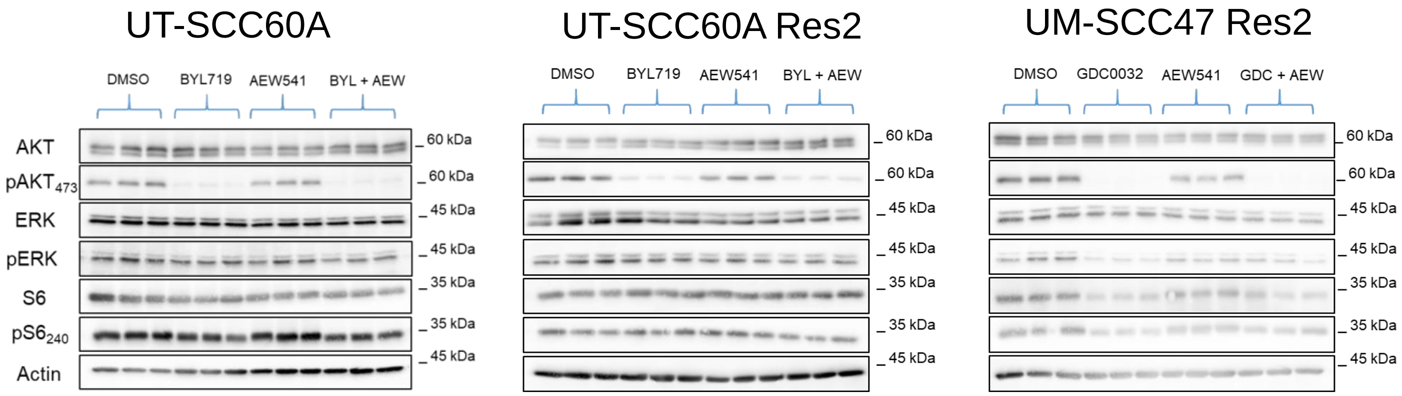

Figure S3: Characterizing of the cellular response of tumor cells to therapy combination. UT-SCC60A sensitive and resistant cells (Res2), and UM-SCC47 resistant cells (Res2) were treated for 4 h with DMSO, BYL719 (2µM) or GDC0032 (500 nM), AEW541 (1 µM) and combination of both drugs, after which cells were lysed. The indicated proteins and phosphorylated proteins were probed using western blot.

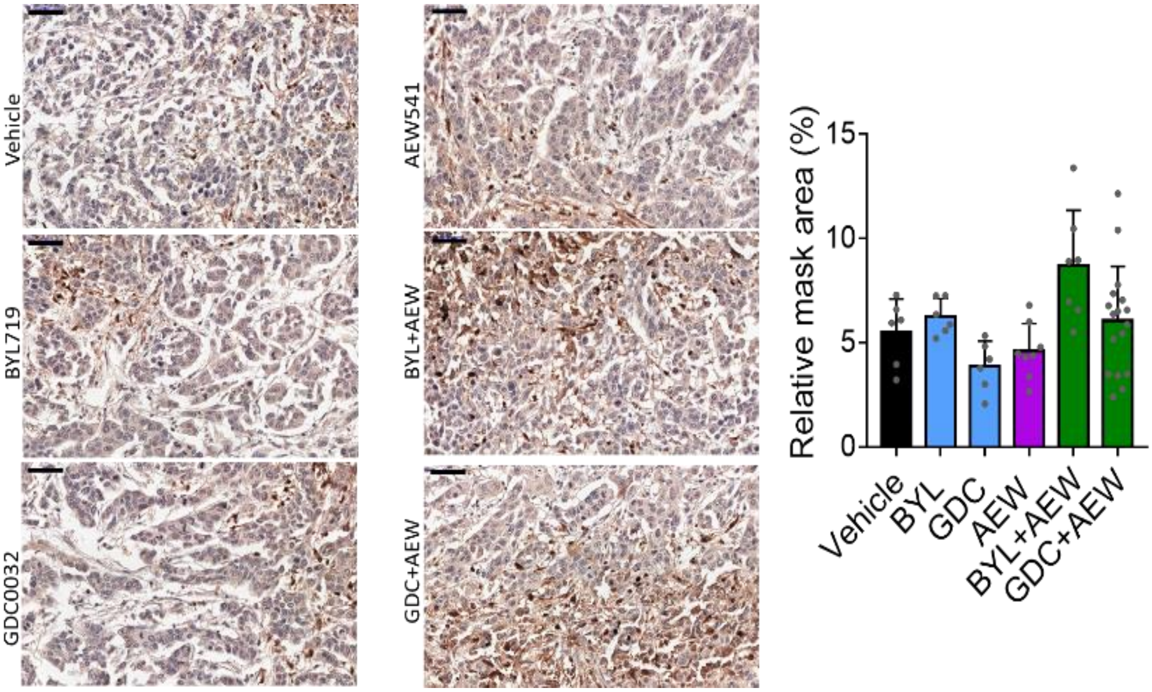

Figure S4: Cleaved Caspase3 staining. PDX^{HPV+} was cultured for 24 h in the presents of DMSO, GDC0032 (500 nM) or BYL719 (2 μM), AEW541 (1 μM) and combinations of drugs as indicated, after which tissue was fixed and analyzed using immunohistochemistry. Representative images of immunohistochemistry staining of Cleaved-Caspase3 are presented with quantification. Scale bar: 50 μm.

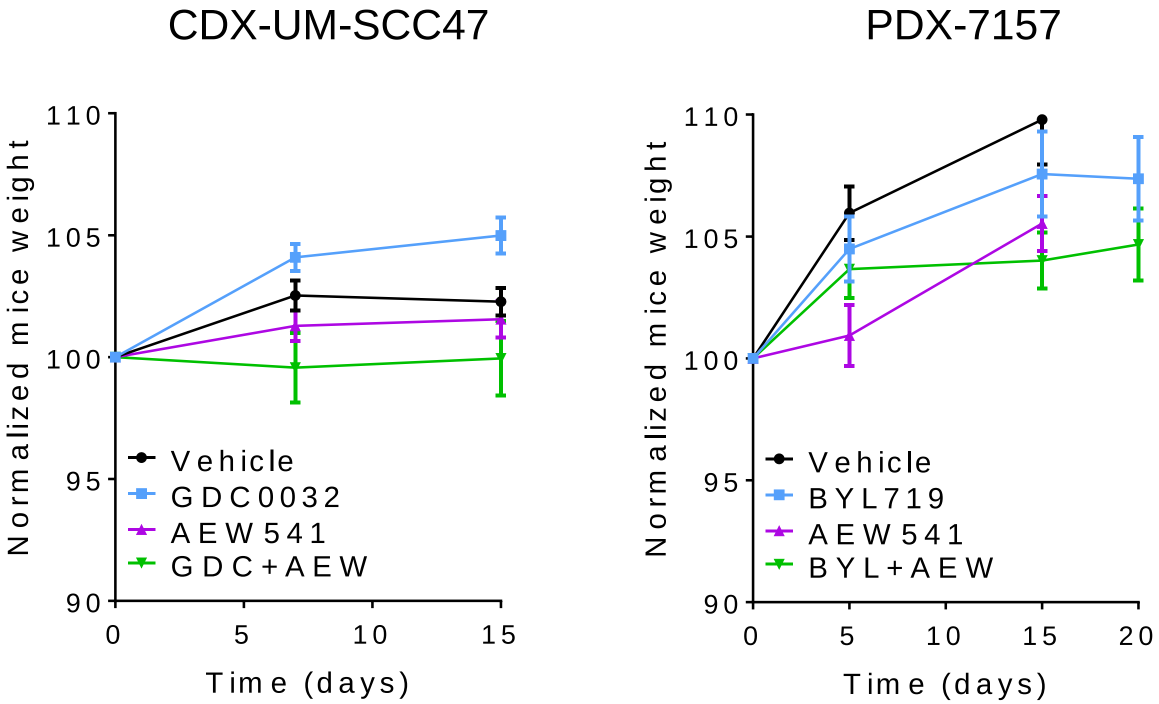

Figure S5: Toxicity of therapy in mice. The weight of NSG mice bearing CDX-UM-SCC47 and PDX-7157 treated as indicated. GDC0032 (10 mg/kg), BYL719 (25 mg/kg), AEW541 (30 mg/kg).

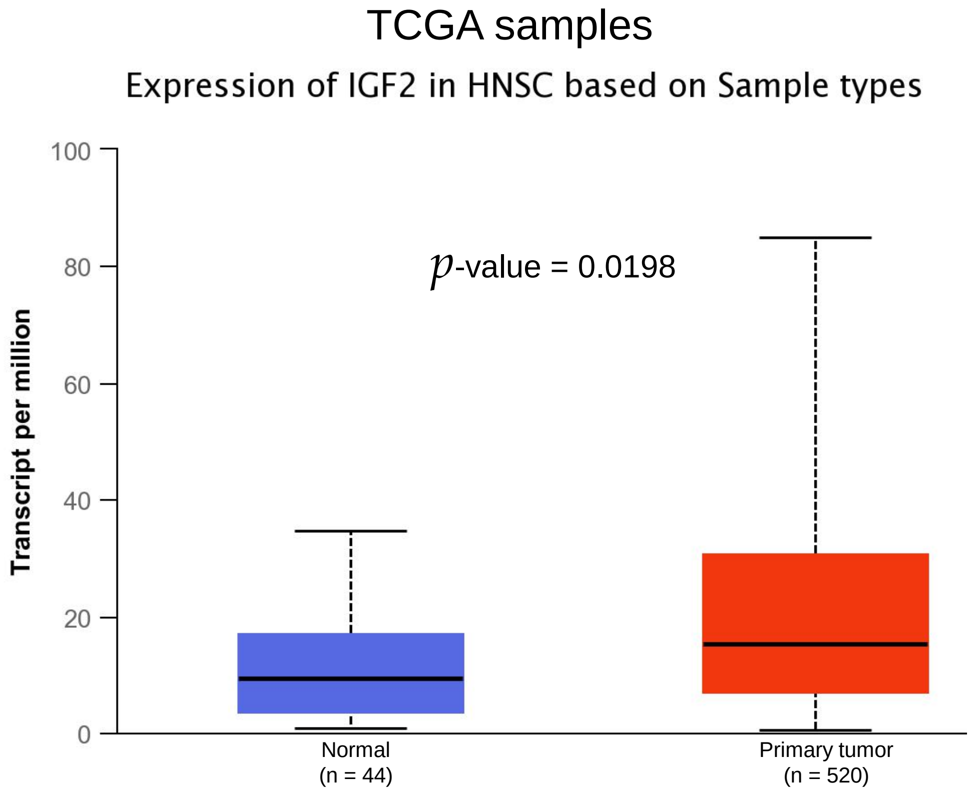

Figure S6: IGF2 expression in HNC patients. TCGA data showing IGF2 expression in HNC patients, compared to normal tissue. Data extracted from HTTP://ulcan.path.uab.edu

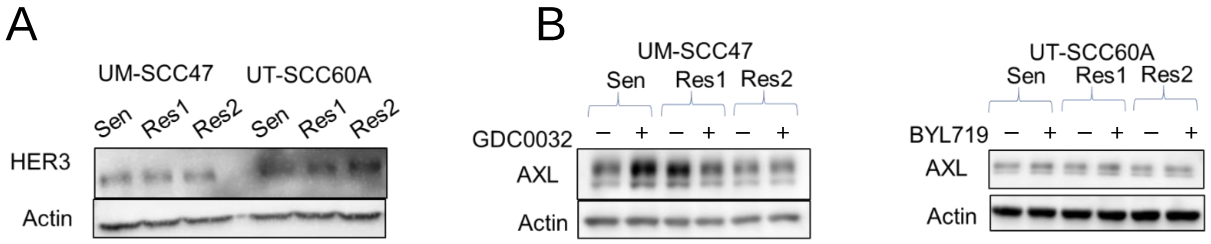

Figure S7: AXL and HER3 expression in sensitive and resistant cells. **(A)** Western blot analysis showing HER3 expression in UM-SCC47 and UT-SCC60A sensitive and resistant cells (Res1 and Res2). **(B)** UM-SCC47 and UT-SCC60A sensitive and resistant cells (Res1 and Res2) were treated with DMSO, GDC0032 (500 nM) or BYL719 (2μM) as indicated. The indicated proteins were measured using *w*estern blot.

Figure S8: Original Western Blot images.

Figure 5A

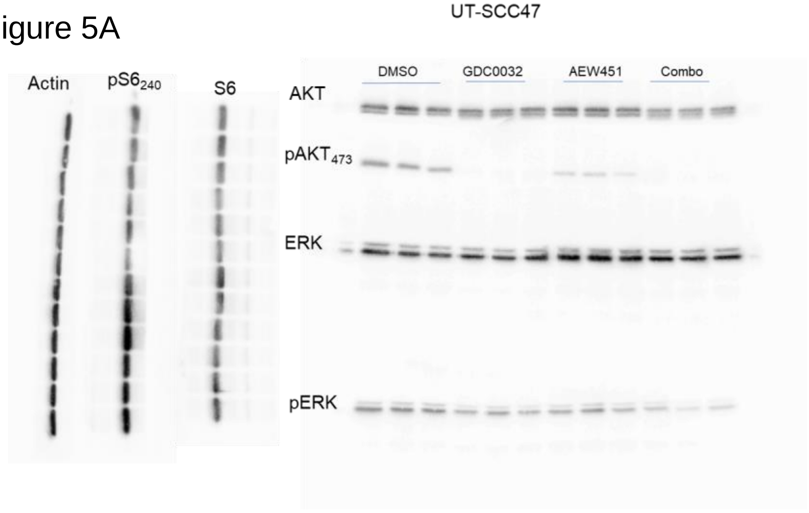

Figure S1B

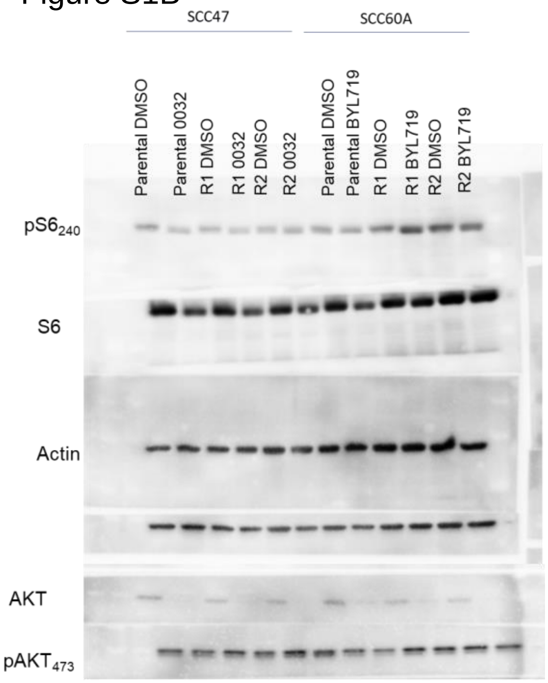

Figure S3

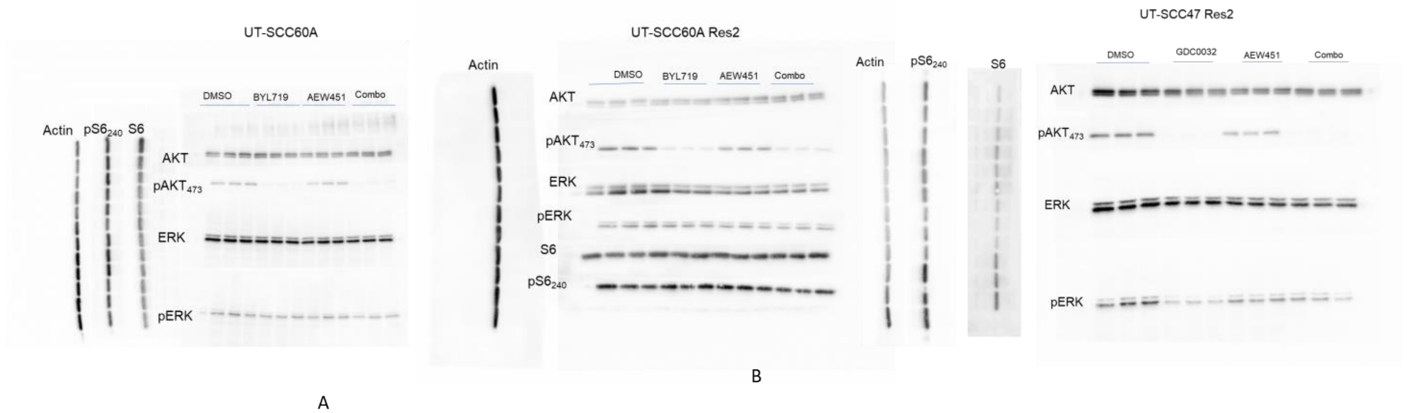

Figure S7

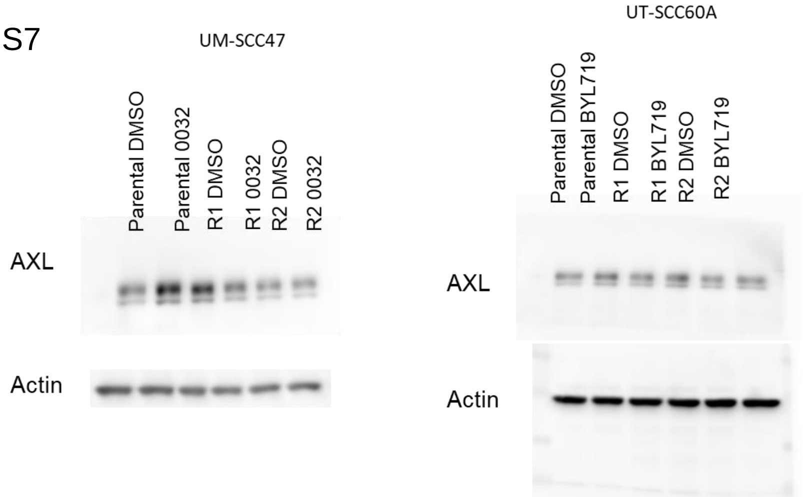

Supplement: Supplementary file 1 [file cancers-13-02250-s001.zip › Final Supplementary Figures.pdf]
